# High temperature inhibited the accumulation of anthocyanin by promoting ABA catabolism in sweet cherry fruits

**DOI:** 10.3389/fpls.2023.1079292

**Published:** 2023-02-13

**Authors:** Yue Tan, Binbin Wen, Li Xu, Xiaojuan Zong, Yugang Sun, Guoqin Wei, Hairong Wei

**Affiliations:** ^1^ Innovation Team of Stone Fruit Breeding and Cultivation, Shandong Institute of Pomology, Tai’an, Shandong, China; ^2^ College of Horticulture Science and Engineering, Shandong Agricultural University, Tai’an, China

**Keywords:** high temperature, sweet cherry, coloring, anthocyanin, ABA

## Abstract

Color is an essential appearance characteristic of sweet cherry (*Prunus avium* L.) fruits and mainly determined by anthocyanin. Temperature plays an important role in the regulation of anthocyanin accumulation. In this research, anthocyanin, sugar, plant hormone and related gene expression were analyzed using physiological and transcriptomic methods in order to reveal the effects of high temperature on fruit coloring and the related mechanism. The results showed that high temperature severely inhibited anthocyanin accumulation in fruit peel and slowed the coloring process. The total anthocyanin content in fruit peel increased by 455% and 84% after 4 days of normal temperature treatment (NT, 24°C day/14°C night) and high temperature treatment (HT, 34°C day/24°C night), respectively. Similarly, the contents of 8 anthocyanin monomers were significantly higher in NT than in HT. HT also affected the levels of sugars and plant hormones. The total soluble sugar content increased by 29.49% and 16.81% in NT and HT, respectively, after 4 days of treatment. The levels of ABA, IAA and GA20 also increased in both the two treatments but more slowly in HT. Conversely, the contents of cZ, cZR and JA decreased more rapidly in HT than in NT. The results of the correlation analysis showed that the ABA and GA20 contents were significantly correlated with the total anthocyanin contents. Further transcriptome analysis showed that HT inhibited the activation of structural genes in anthocyanin biosynthesis as well as the repression of *CYP707A* and AOG, which dominated the catabolism and inactivation of ABA. These results indicate that ABA may be a key regulator in the high-temperature-inhibited fruit coloring of sweet cherry. High temperature induces higher ABA catabolism and inactivation, leading to lower ABA levels and finally resulting in slow coloring.

## 1 Introduction

Sweet cherry (*Prunus avium* L.) is one of the earliest fresh fruits available in northern China and has high economic value. The fruit is deeply loved by people for its bright color and rich nutrition, conferred by a variety of carbohydrates, proteins, and vitamins as well as iron, calcium, potassium and other essential nutrients and minerals (Li et al., 2010; Prinsi et al., 2016; Gonçalves et al., 2021). The coloring of sweet cherry peel is a major factor affecting fruit quality and largely determines its market value. Anthocyanin is responsible for the red color of sweet cherry peel, and its content directly affects the color of fruits.

The anthocyanin biosynthetic pathway has been studied in many plants, and related genes have been identified (Petroni and Tonelli, 2011; Zhai et al., 2014; Dai et al., 2022). The regulation of anthocyanin biosynthesis mainly involves a number of structural genes such as phenylalanine ammonia-lyase (*PAL*), 4-coumarate-CoA ligase (*4CL*), chalcone synthase (*CHS*), chalcone isomerase (*CHI*), flavanone-3-hydroxylase (*F3H*), dihydroflavonol 4-reductase (*DFR*), anthocyanidin synthase (*ANS*), UDP-glucose flavonoid 3-O-glucosyltransferase (*UFGT*) and leucoanthocyanidin dioxygenase (*LDOX*), as well as the MYB-bHLH-WD40 (MBW) transcription complex (Kim et al., 2006; Albert et al., 2014; Xue et al., 2016; Moglia et al., 2020). MYB10 promotes anthocyanin accumulation by activating the promoters of *DFR* and *UGFT* (Wang et al., 2013; Zhai et al., 2016). The coexpression of basic helix-loop-helix (bHLH) and MYB10 proteins activates the transcription of structural genes (*CHS*, *DFR* and *UFGT*) (Rahim et al., 2014). WD40 domain-containing protein (WD40) is associated with the stabilization of the MBW complex, and the interaction between TTG1 (a WD40 protein) and bHLH plays an important role in promoting anthocyanin biosynthesis (An et al., 2012).

Anthocyanin accumulation is affected by multiple biotic and abiotic factors, such as nutrition, temperature, light and wounding (Weiss, 2000; Lin-Wang et al., 2011; Günther et al., 2022). Low temperature can induce anthocyanin biosynthesis and the expression of related genes, while high temperature accelerates anthocyanin degradation (Rowan et al., 2009; Rehman et al., 2017) and is unfavorable for anthocyanin synthesis (Crifò et al., 2012; Ryu et al., 2020). In rose (*Rosa hybrida*), high temperature (39 °C/18 °C) decreases the anthocyanin content in flowers by inhibiting the transcriptional levels of CHS and DFR (Dela et al., 2003). In *Arabidopsis thaliana*, high temperature reduces anthocyanin accumulation by decreasing the expression of genes and transcription complexes related to anthocyanin synthesis and increasing the expression of the anthocyanin repressors *AtMYB3*, *AtMYB6* and *AtMYBL2* (Rowan et al., 2009).

Plant hormones are important regulators of anthocyanin biosynthesis. Exogenous application of abscisic acid and jasmonic acid promotes anthocyanin accumulation in multiple fruits (Shen et al., 2014; An et al., 2015; An et al., 2018; Wu et al., 2020; Gao et al., 2021). Gibberellins negatively regulate low-temperature-induced anthocyanin accumulation in Arabidopsis (Zhang et al., 2011). Cytokinin (Ji et al., 2015; Wang et al., 2019), auxin (Mori et al., 1994; Wang et al., 2020) and brassinolide (Zheng et al., 2018) are also effective in regulating anthocyanin biosynthesis in multiple fruits. Among these plant hormones, abscisic acid mediates anthocyanin biosynthesis in sweet cherry, and MYBA is involved in the process (Shen et al., 2014). Although the effect of abscisic acid on anthocyanin biosynthesis has been widely studied, the molecular mechanism by which the interaction between high temperature and abscisic acid regulates anthocyanin biosynthesis is still unknown.

Color changes occur as an adaptation to changes in the external environment during the development of plant tissue (Oren-Shamir, 2009). The coloring period of sweet cherry in northern China is from late April to early May. The weather in this period is atypical, and the temperature usually increases sharply, which may have a profound impact on fruit coloring. This phenomenon will be a common problem in sweet cherry production, which directly affects the color and quality of fruits under global warming. Although the effects of temperature on anthocyanin biosynthesis have been analyzed in fruit species such as apple (Fang et al., 2019), grape (Yang et al., 2020) and pear (Zhai et al., 2016), few related studies have been conducted on sweet cherry, and the associated mechanism remains unclear.

In the present study, we found that high-temperature-treated sweet cherry peels accumulated less anthocyanin than normal-temperature-treated sweet cherry peels. Further study revealed that ABA may play a key role in the high-temperature-inhibited fruit coloring of sweet cherry. This study provides a new approach for studying the effect of temperature on anthocyanin accumulation in sweet cherry peel and provides a theoretical basis for improving the quality and commercial value of sweet cherry.

## 2 Materials and methods

### 2.1 Plant materials and treatment

The branches of ‘Tieton’ sweet cherry (*Prunus avium* L. Tieton) were collected at the beginning of coloring from the experimental orchard of the Shandong Institute of Pomology (36°21′N, 117°12′E; Tai’an China). At 25 days after flowering, fruiting branches showing similar growth were selected, and equal numbers of leaves from each branch were brought back to the laboratory. The collected branches were inserted into a glass bottle containing sterile water and placed in an artificial climate chamber for different temperature treatments. The high temperature treatment (HT) was set at 24°C (night)/34°C (day), and the normal temperature treatment (NT) was set at 14°C (night)/24°C (day). The light intensity was set at 20,000 lx, and the light cycle was 8 hours dark/16 hours light. The relative humidity was approximately 70%. After 4 days of treatment, the peel of sweet cherry was cut, immediately frozen in liquid nitrogen and stored at -80°C until use. The abbreviations used for the samples are as follows: BT represents samples collected before treatment, NT represents the normal temperature treatment and HT represents those subjected to high temperature treatment. Finally, three biological replicates of three samples each (9 samples in total) were used for further analysis.

### 2.2 Measurement of total anthocyanin and anthocyanin components

The extraction of total anthocyanin was based on Harborne’s method. The sweet cherries collected at 25 days after flowering were placed in a phytotron (light intensity 20000 lx, humidity 70%) for four days. Samples of approximately 1 g of the HT (24°C night/34°C day)- and NT (14°C night/24°C day)-treated sweet cherries were incubated in 10 ml of 1% (v/v) HCl-95% ethanol at 24°C for 24 hours. The absorbance was measured at 530, 620, and 650 nm with a UV−vis spectrophotometer (UV-2600, SHIMADZU, Chengdu, China).

The analysis of anthocyanin components was conducted by MetWare Biotechnology Co., Ltd. (Wuhan, China) using a UPLC−MS/MS platform. The standard procedures from the separation of substances by chromatography to identification by mass spectrometry have been fully described by Yang et al. (Mori et al., 2007), Zhang, et al. (Zhang et al., 2019a) and Zhang, et al. (Zhang et al., 2019b). The mass spectrometry data were processed with Analyst 1.6.3 software (AB Sciex, Ontario, Canada). The total ion current (TIC) diagrams of the various quality normal (QC) samples were overlapped to determine the repeatability of the metabolic extraction and detection results. The metabolite content data were normalized according to unit variance scaling. A multivariate statistical analysis method with supervised pattern recognition, partial least square discriminant analysis (PLS-DA), was used to maximize the differentiation between groups. Based on the orthogonal projections to latent structures discriminant analysis (OPLS-DA) results, the differentially accumulated metabolites were preliminarily screened using variable importance in projection (VIP). Metabolites exhibiting a fold change of ≥2 or ≤0.5 and VIP≥1 were considered to present significant differences between groups. The KEGG database (Kanehisa et al., 2004) was used to annotate the differentially abundant metabolites.

### 2.3 Measurement of soluble sugar components, total acid and pH

The soluble sugar content was determined by high-performance liquid chromatography (Waters 510, PA, USA) with an RID-10 differential detector. The chromatographic column used for measuring fructose, glucose and sorbitol was a carbohydrate Ca^2+^ column (250 mm * 4.6 mm); the mobile phase was 1000 ml of water; the column temperature was 80°C; the flow rate was 0.4 ml/min; and the injection volume was 10 µl. The chromatographic column used for measuring sucrose and galactose was a Carbomi xH-NP column (300*7.8 mm), the mobile phase was 1000 ml of water and 136 µl of sulfuric acid, the column temperature was 55°C, the flow rate was 0.6 ml/min, and the injection volume was 10 µl.

The determination of the total acid content was performed with reference to the OIV method (OIV 2012). The pH was measured with a PH400 desktop pH meter (PH400, Alalis, USA).

### 2.4 Measurement of plant hormones

Approximately 50 mg of each sample was ground in liquid nitrogen and dissolved in 1 mL methanol/water/formic acid (15:4:1, V/V/V). Ten microliters of internal standard mixed solution (100 ng/mL) was added as an internal standard. The mixture was vortexed for 10 minutes and centrifuged for 5 min (12000 r/min, and 4°C). The supernatant was then transferred into clean microtubes, evaporated to dry, dissolved in 100 μL 80% methanol (V/V) and filtered through a 0.22 μm membrane filter for further analysis.

The analysis was performed using a UPLC−ESI−MS/MS system (UPLC, ExionLC™ AD, SCIEX, USA; MS, Applied Biosystems 6500 Triple Quadrupole, USA). The analytical conditions were as follows: LC: column, Waters ACQUITY UPLC HSS T3 C18 (100 mm×2.1 mm, 1.8 µm); solvent system: water with 0.04% acetic acid (A), acetonitrile with 0.04% acetic acid (B); gradient program: started at 5% B (0-1 min), increased to 95% B (1-8 min), 95% B (8-9 min), and finally decreased to 5% B (9.1-12 min); flow rate: 0.35 mL/min; temperature: 40°C; and injection volume: 2 μL.

Linear ion trap and triple quadrupole scans were acquired on a triple quadrupole–linear ion trap mass spectrometer (QTRAP), QTRAP^®^ 6500+ LC−MS/MS System, controlled by Analyst 1.6.3 software (SCIEX, USA). The ESI source operation parameters were as follows: ion source, ESI+/-; source temperature 550°C; ion spray voltage (IS) 5500 V (positive), -4500 V (negative); curtain gas (CUR) was set at 35 psi. Plant hormones were analyzed using scheduled multiple reaction monitoring (MRM). Multiquant 3.0.3 software (Sciex) was used to quantify all metabolites. Mass spectrometer parameters, including the declustering potentials (DP) and collision energies (CE) for individual MRM transitions, were performed with further DP and CE optimization. A specific set of MRM transitions was monitored for each period according to the metabolites eluted within this period.

### 2.5 RNA extraction, identification and transcriptome analysis

Total RNA was extracted from 3 biological replicates of sweet cherry peel using the RNAprep Pure Plant Kit (for polysaccharide- and polyphenolic-rich plants) (DP441, TIANGEN, Beijing, China). The quality of the RNA was measured using 1% agarose gel electrophoresis (18S and 28S) to ensure that the RNA was not contaminated or degraded. Nanodrop (IMPLEN, CA, USA), Qubit 2.0 (Life Technologies, CA, USA) and Agilent 2100 (Agilent Technologies, CA, USA) systems were used to detect the purity, concentration and integrity of the RNA samples to ensure the use of qualified samples for transcriptome sequencing.

PCR enrichment was conducted to construct the cDNA library after the samples were qualified. Qubit 2.0 (Life Technologies, CA, USA) and Agilent 2100 (Agilent Technologies, CA, USA) systems were used to determine the library concentration and insert size, respectively. qRT−PCR was used to accurately quantify the effective concentration of the library. The library was then sequenced on the Illumina HiSeq platform, and 150-bp paired-end reads were obtained. Based on the use of sequencing-by-synthesis technology, large amounts of high-quality data were obtained, and most of the base-pair quality values reached or exceeded Q30. The transcriptome data were compared with the reference genome sequence (GenBank: JAAOZG010000000) by using the HISAT2 system (Kim et al., 2015). After the comparative analysis was completed, the mapped reads were assembled and quantified using StringTie.

BLAST software was used to compare the extracted new genes with the NR, Swiss-Prot, GO, COG, KOG, Pfam and KEGG databases. The KEGG orthology analysis of the new genes was performed by using KOBAS2.0 (Xie et al., 2011). Fragments per kilobase of transcript per million mapped reads (FPKM) (Florea et al., 2013) values were used as an indicator to measure the expression levels of the transcripts or genes. DESeq was used for differential expression analysis between different sample groups (Wang et al., 2010). Fold change (FC) ≥ 2 or ≤ 0.5 and a false discovery rate (FDR) < 0.01 were used as the criteria for the identification of differentially expressed genes (DEGs). The DEGs identified between samples were subjected to GO and KEGG analyses with the R package.

### 2.6 Quantitative real-time PCR (qRT−PCR) analysis

To verify the results of the transcriptome analysis, we selected 13 genes that were significantly enriched in the anthocyanin biosynthetic pathway for RT−PCR verification. First-strand cDNA was synthesized by using a cDNA reverse transcription kit (RR047A, TaKaRa, Dalian, China). The primers used in these steps are listed in **Supplementary Material S1**. qRT−PCR was performed on a CFX Connect™ real-time PCR system (Bio-Rad, Hercules, CA, USA) using SYBR^®^ Premix Ex Taq™ (Tli RNaseH Plus) (RR420A, TaKaRa, Dalian, China). The reaction system included 12.5 μL of SYBR^®^ Premix Ex Taq (2 ×), 1 μL of each forward and reverse primer, 1 μL of cDNA, and ddH_2_O to 25 μL. Three technical replicates were analyzed for each sample. The qRT−PCR conditions were as follows: 40 cycles of predenaturation at 95°C for 30 s, denaturation at 95°C for 5 s, and annealing at 60°C for 30 s. A fluorescence curve and a melting curve were obtained, and relative expression levels were calculated using the 2^-△△CT^ method.

### 2.7 Statistical analysis

Statistical analysis was performed using the software Microsoft Excel 2010. Graphs were obtained from GraphPad Prism 6.01. The values in each figure were the mean ± SD of three replicates. Significant differences analysis was performed with SPSS 19.0 using Duncan’s test, and differences at p < 0.05 were labeled for the statistical tests. Pearson’s correlation analysis was conducted using SPSS 19.0 with a critical level of p < 0.05.

## 3 Results

### 3.1 High temperature inhibited anthocyanin accumulation in sweet cherry peel

**Figure 1:**
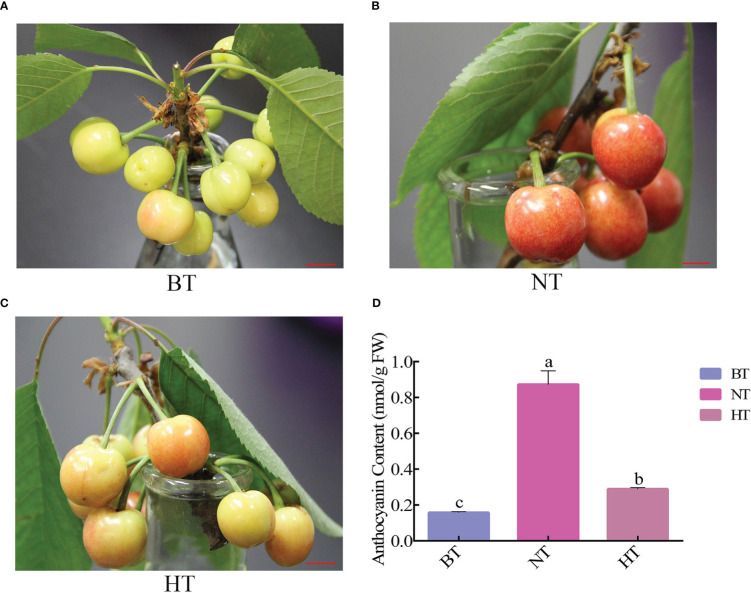
High temperature represses anthocyanin accumulation in sweet cherry peel. **(A)** Phenotype of sweet cherry before treatment (BT). **(B)** Phenotype of sweet cherry treated at normal temperature [NT, 14°C (night)/24°C (day)]. **(C)** Phenotype of sweet cherry treated with high temperature [HT, 24°C (night)/34°C (day)]. **(D)** The anthocyanin content in different temperature treatment groups. The different letters indicate significant differences among different treatments according to Duncan’s test (p < 0.05).

HT inhibited anthocyanin accumulation in fruit peel and severely delayed the coloring process of sweet cherry fruits. The fruits turned bright red from green yellow after 4 days in NT, while the fruits in HT only colored slightly (**Figures 1A–C**). The total anthocyanin contents in fruit peel increased by 455% and 84% in NT and HT, respectively (**Figure 1D**). The final total anthocyanin level in HT was only approximately 1/3 of that in NT.

**Figure 2:**
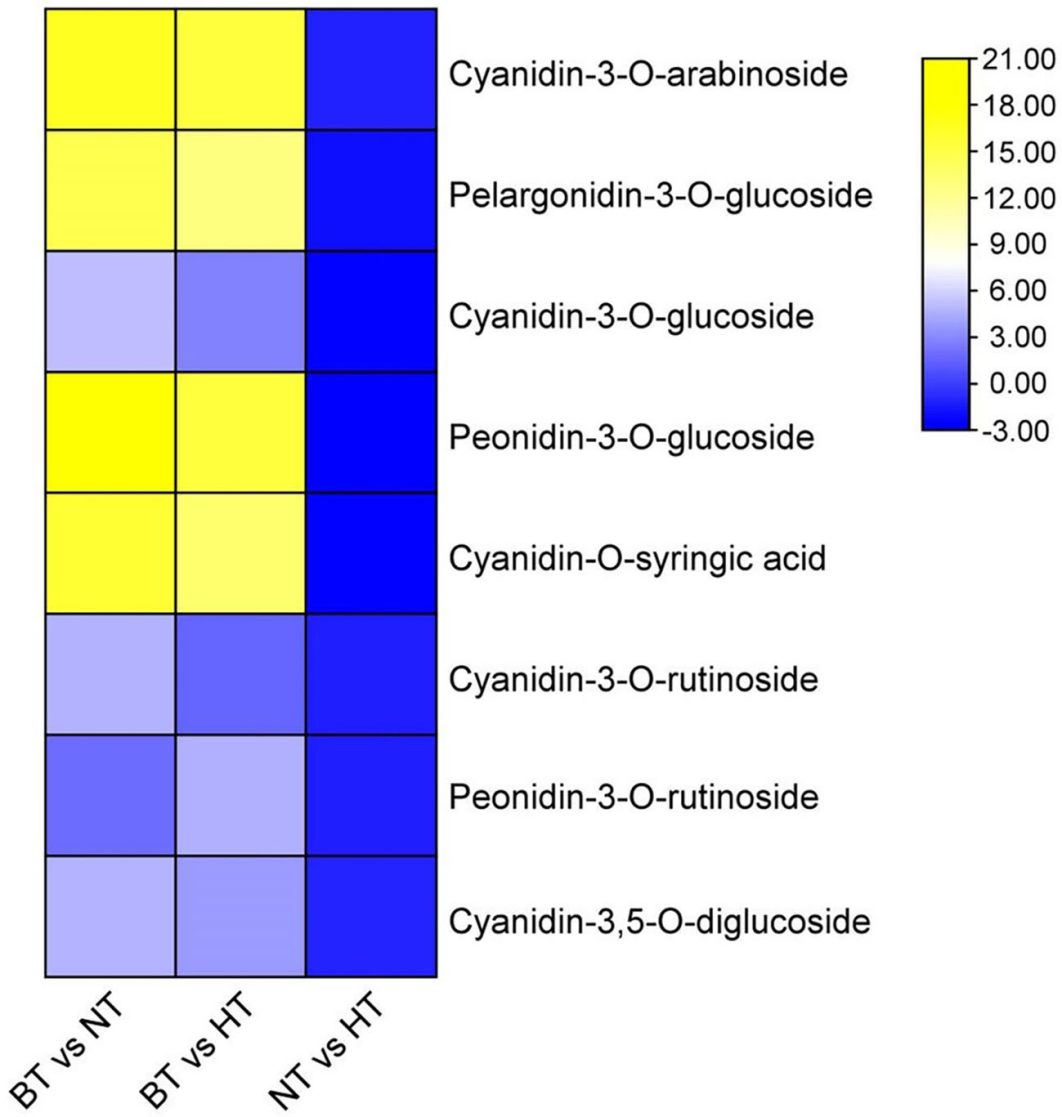
Differential accumulation of anthocyanin monomers in sweet cherry peel. Anthocyanin monomers were visualized as a heat map based on log_2_(fold change) obtained from comparisons among different treatments.

The variety and level of anthocyanin monomers were further analyzed using UPLC−MS/MS to reveal details of the anthocyanin change. A total of 511 metabolites, including 48 differentially accumulated metabolites (DAMs), were detected in NT and HT. The DAMs mainly included flavonoids, phenolic acids, lipids and alkaloids (**Supplementary Material S2**). In the DAMs, most lipids and flavonoids had higher abundance NT, while most phenolic acids and alkaloids had higher abundance in HT. Among the detected metabolites, 21 were identified to be anthocyanin monomers, and 8 of them were significantly less abundant in HT than in NT. The contents of the 8 anthocyanin monomers ranged from 18.01% to 49.06% of those in NT (**Figure 2**). The levels of the remaining 13 anthocyanin monomers showed no difference between HT and NT. The metabolic data are deposited in the figshare database (https://figshare.com/articles/dataset/metabolic_data_of_sweet_cherry/13571306).

### 3.2 High temperature inhibited sugar accumulation in sweet cherry peel

**Figure 3:**
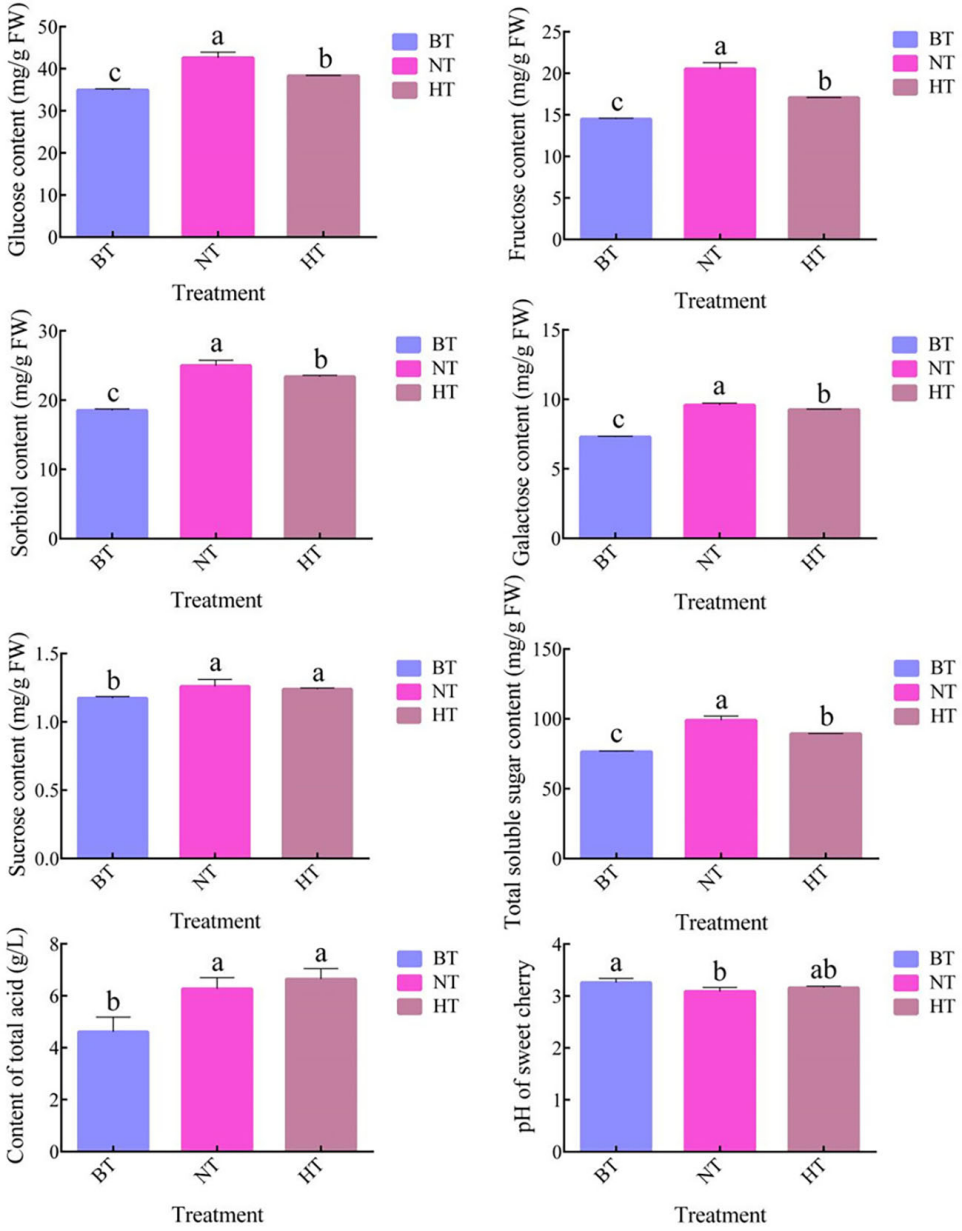
The soluble sugar content, total acid content and pH of sweet cherry peel in different treatments. The values represent the means ± SDs, n = 3. The different letters indicate significant differences among different treatments according to Duncan’s test (p < 0.05).

In accordance with the change in anthocyanin, the accumulation of sugar in fruit peel in HT was less than that in NT (**Figure 3**). The total soluble sugar content increased by 29.49% and 16.81% in NT and HT, respectively, after 4 days of treatment. The total soluble sugar content of sweet cherry peel in the HT group was 9.79% lower than that in the NT group. Further analysis showed that the difference mainly came from glucose, fructose, sorbitol and galactose. The contents of the 4 sugars were 10.04%, 16.83% 6.45% and 3.40% lower in HT than in NT, respectively. Sucrose accounted for no more than 2% of the total soluble sugar and showed no significant difference in HT and NT. No significant difference in total acid content or pH was found between HT and NT (**Figure 3**).

### 3.3 High temperature changed hormone levels in sweet cherry peel

**Figure 4:**
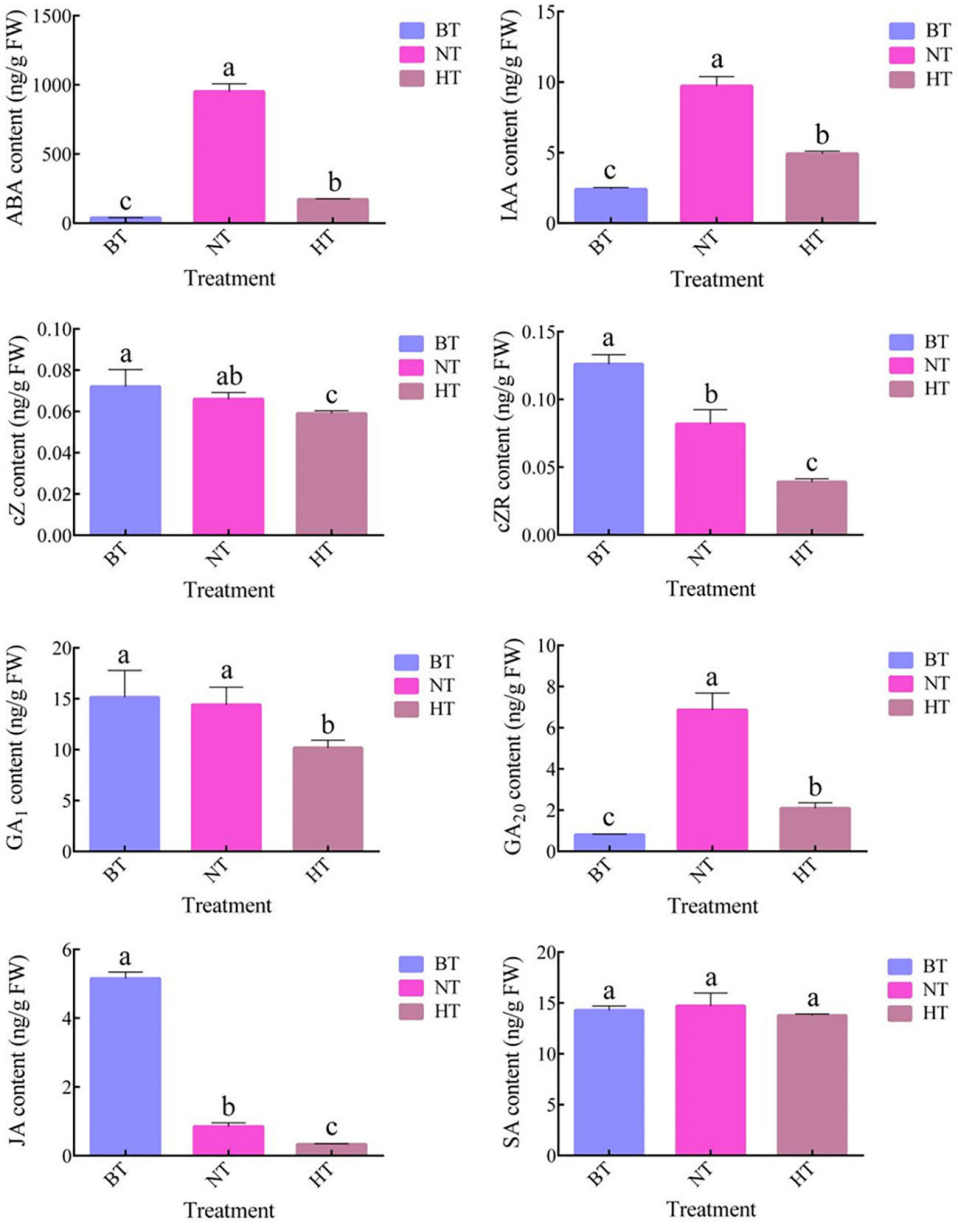
The contents of plant hormones in sweet cherry peel under different treatments. The values represent the means ± SDs, n = 3. The different letters indicate significant differences among different treatments according to Duncan’s test (p < 0.05).

Plant hormones are important regulators of anthocyanin biosynthesis in fruits. Our research showed that high temperature led to dramatic changes in plant hormone levels in sweet cherry fruit peel (**Figure 4**). The levels of abscisic acid (ABA), auxin (IAA) and gibberellic acid 20 (GA_20_) increased in both the NT and HT treatments, while the contents of cis-zeatin (cZ), cis-zeatin riboside (cZR) and jasmonic acid (JA) showed downward trends. Interestingly, the contents of the six compounds were all lower in HT than in NT. Notably, the level of ABA, an important regulator of anthocyanin biosynthesis in sweet cherry (Shen et al., 2014), was only 18.07% in HT compared with NT. The levels of GA_1_ and salicylic acid (SA) showed no significant difference among samples from BT, NT and HT. Some common hormones, including trans-zeatin (tZ), GA_3_, GA_4_ and GA_7,_ were not detected in the samples.

### 3.4 Correlation analysis between anthocyanin content and other physiological parameters

Pearson’s correlation analysis was further conducted to screen potential physiological factors affecting anthocyanin biosynthesis in sweet cherry peels (**Supplementary Material S3**). The results showed that the anthocyanin level was significantly correlated with the ABA and GA_20_ contents. High correlation coefficients also existed between the anthocyanin levels and glucose, fructose, sorbitol, total sugar and IAA contents regardless of the significance analysis.

### 3.5 Comparative transcriptome analysis of sweet cherry peel under different treatments

**Figure 5:**
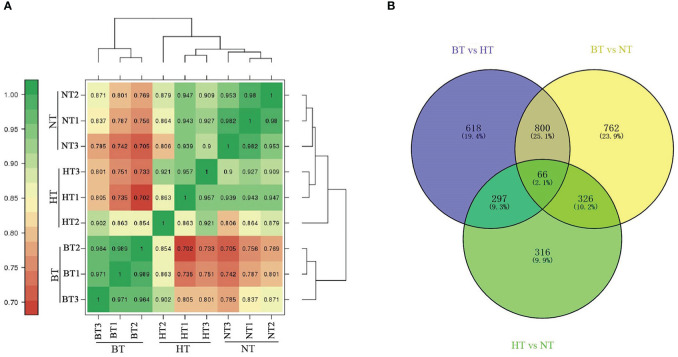
Analysis of DEGs in the transcriptome. **(A)** Heatmap of the correlation coefficients of different temperature treatment groups. **(B)** Venn diagram of DEGs.

Clean data (79.85 Gb) were generated after mRNA sequencing for 9 samples from BT, HT and NT (including 3 biological replicates). A total of 39,695 transcripts were obtained by processing the sequencing reads. The expression levels were calculated using the FPKM method (Trapnell et al., 2010). Pairwise Pearson’s correlation analysis showed high consistency of the biological replicates (**Figure 5A**). The RNAseq data generated for this study is available in the GenBank database (http://www.ncbi.nlm.nih.gov/bioproject/678548).

Differentially expressed genes (DEGs) were further identified using the DESeq R package (1.18.0) with the criteria of |log_2_FoldChange| ≥ 1 and FDR < 0.01. A total of 1,781 and 1,954 DEGs were obtained from BT vs. HT and BT vs. NT, respectively, while only 1,005 DEGs were obtained from NT vs. HT. The Venn diagram showed that 866 DEGs were shared in BT vs. HT and BT vs. NT, accounting for more than 40% of the DEGs of each group (**Figure 5B**). However, the numbers in BT vs. HT and NT vs. HT (363 shared DEGs) or BT vs. NT and NT vs. HT (392 shared DEGs) were much lower. The statistical data of DEGs in each group are shown in **Supplementary Material S4**.

### 3.6 DEGs involved in anthocyanin biosynthesis

The DEGs involved in anthocyanin biosynthesis and its upstream regulation were further screened through KEGG analysis and manually. These DEGs mainly included structural genes involved in anthocyanin biosynthesis, genes encoding the MBW (MYB-bHLH-WD40) complex and genes participating in plant hormone metabolism and plant hormone signal transduction. **Table 1** shows the expression of structural genes involved in anthocyanin biosynthesis. Compared with BT, the expression of most genes was significantly upregulated in NT, while only 4 of them were upregulated in HT. The expression of 2 *PAL* genes and 1 *4CL* genes was upregulated in both NT and HT, while *UFGT* was upregulated only in HT. The expression of *CHS*, *CHI*, *DFR* and *ANS* was significantly lower in HT than in NT.

**Table 1:** Differentially expressed structural genes involved in anthocyanin biosynthesis and downregulated *MYB*, *bHLH* and *WD40* genes in NT vs. HT.

Class	Gene name	Gene ID	Log_2_ (fold change)		
			BT vs NT	BT vs HT	NT vs HT
	*PAL*	gene_Pav_co4071347.1_g010.1.mk	4.21	3.36	/
		gene_Pav_sc0000711.1_g010.1.mk	4.11	3.22	/
		gene_Pav_sc0000084.1_g560.1.mk	-2.64	/	/
	*C4H*	gene_Pav_sc0000206.1_g540.1.mk	1.57	/	/
	*4CL*	gene_Pav_sc0000636.1_g260.1.mk	3.31	2.74	/
		gene_Pav_sc0000351.1_g340.1.mk	-1.10	/	/
	*CHS*	gene_Pav_sc0000045.1_g280.1.mk	3.01	/	-2.46
	*CHI*	gene_Pav_sc0007510.1_g020.1.mk	1.24	/	-1.16
		gene_Pav_sc0000554.1_g2230.1.mk	1.56	/	-1.75
		gene_Pav_sc0005746.1_g030.1.mk	/	-1.11	-1.89
	*F3H*	gene_Pav_sc0000044.1_g550.1.mk	1.83	/	/
		gene_Pav_sc0000030.1_g1340.1.mk	-6.18	-7.48	/
	*F3’H*	gene_Pav_sc0000877.1_g1900.1.br	2.48	/	/
	*DFR*	gene_Pav_sc0002208.1_g840.1.mk	3.10	/	-1.64
	*ANS*	gene_Pav_sc0000107.1_g100.1.mk	1.26	/	-2.01
	*UFGT*	gene_Pav_sc0001126.1_g120.1.br	/	3.88	2.46
*MYB*	*REV 8-like*	gene_Pav_sc0003845.1_g090.1.mk	2.19	/	-1.92
	*DIV-like*	gene_Pav_sc0000580.1_g150.1.mk	/	-1.23	-1.52
*bHLH*	*bHLH13*	gene_Pav_sc0000586.1_g190.1.mk	1.31	-1.04	-2.36
	*bHLH94-like*	Prunus_avium_newGene_3792	/	/	-2.64
	*bHLH-145*	gene_Pav_sc0000257.1_g830.1.mk	/	/	-1.03
*WD40*	*LUH-like*	gene_Pav_sc0001963.1_g370.1.mk	/	/	-1.12
	*CDC20.1*	gene_Pav_sc0001557.1_g120.1.br	1.88	/	-1.34

PAL, phenylalanine ammonia-lyase; C4H, cinnamate 4-hydroxylase; 4CL, 4-coumarate-CoA ligase; CHS, chalcone synthase; CHI, chalcone isomerase; F3H, flavanone-3-hydroxylase; F3’H, flavonoid 3’-hydroxylase; DFR, dihydroflavonol 4-reductase; ANS, anthocyanidin synthase; UFGT, UDP-glucose flavonoid 3-O-glucosyltransferase; REV, REVEILLE; DIV, DIVARICATA; LUH, LEUNIG_HOMOLOG; CDC, cell division cycle. “/” denotes that no significant difference was observed.

The expression of anthocyanin biosynthesis-associated structural genes is regulated by the MYB-bHLH-WD40 complex comprising MYB transcription factor, bHLH transcription factor and WD40 protein (Jaakola, 2013). Considering the downregulation of these structural genes in HT compared with NT, the downregulated genes belonging to the *MYB*, *bHLH* and *WD40* classes in NT vs. HT were screened to explore the upstream regulators. A total of 7 genes were obtained (**Table 1**), including 2 in the *MYB* class, 3 in the *bHLH* class and 2 in the *WD40* class. The 4 genes belonging to the *MYB* and *WD40* classes were not involved in anthocyanin biosynthesis according to the present literature. The expression levels of *bHLH13*, *bHLH94-like* and *bHLH-145* were significantly lower in HT than in NT. Notably, the expression of *bHLH13* was upregulated in NT but downregulated in HT.

### 3.7 DEGs involved in ABA metabolism and signal transduction

ABA has been proven to be an important regulator of anthocyanin biosynthesis in sweet cherry (Ren and Leng, 2010; Shen et al., 2014). The DEGs involved in ABA metabolism and signal transduction were further screened considering the significant correlation between ABA levels and anthocyanin levels in this research. A total of 4 DEGs were identified participating the biosynthesis and catabolism of ABA (**Table 2**). β-carotene 3-hydroxylase (BCH) and zeaxanthin epoxidase (ZEP) catalyzed the biosynthesis of violaxanthin (an intermediate products of ABA biosynthesis) from β-carotene (Hieber et al., 2000; Xia et al., 2022). Both *BCH2* and ZEP showed lowed expression level in HT than in NT (**Table 2**). Cytochrome P450 707A (CYP707A), also known as ABA 8’-hydroxylase, is the key enzyme in ABA catabolism (Saito et al., 2004). Abscisate *β*-glucosyltransferase (AOG) catalyzes the glycosylation and inactivation of ABA (Xu et al., 2002). The expression levels of *CYP707A* and *AOG* were both lower in NT than in BT but higher in HT than in NT (**Table 2**). The expression levels of the 4 genes are consistent with the difference in ABA levels among the 3 treatments (**Figure 4**). Further analysis showed that the expression of most genes involved in ABA signal transduction showed little difference between NT and HT.

**Table 2:** Expression of DEGs involved in the biosynthesis, catabolism and signal transduction of plant hormones.

Pathway	Gene name	Gene ID	Log_2_ (fold change)		
			BT vs NT	BT vs HT	NT vs HT
ABA biosynthesis	*BCH*	gene_Pav_sc0001405.1_g1500.1.mk	1.76	/	-1.46
	*ZEP*	gene_Pav_sc0000071.1_g630.1.mk	/	/	-1.00
ABA catabolism	*CYP707A*	gene_Pav_sc0000071.1_g720.1.mk	-8.65	-2.65	5.98
	*AOG*	gene_Pav_sc0001126.1_g270.1.br	-1.30	/	2.38
ABA signal transduction	*PYL*	gene_Pav_sc0001341.1_g250.1.mk	1.35	2.12	/
		gene_Pav_sc0000591.1_g120.1.mk		1.29	/
	*PP2C*	gene_Pav_sc0000129.1_g370.1.mk	1.89	1.00	/
		gene_Pav_sc0000212.1_g830.1.mk	2.00	/	/
		gene_Pav_sc0000689.1_g440.1.mk	1.17	/	/
		gene_Pav_sc0001335.1_g050.1.mk	1.58	/	/
	*SNRK2*	gene_Pav_sc0000549.1_g120.1.mk	1.37	/	-1.61
		gene_Pav_sc0000639.1_g120.1.mk	1.20	1.10	/
	*ABF*	gene_Pav_sc0000363.1_g920.1.mk	1.53	1.02	/
		gene_Pav_sc0000852.1_g810.1.mk	1.64	1.42	/

BCH, β-carotene 3-hydroxylase; ZEP, zeaxanthin epoxidase; CYP707A, cytochrome P450 707A (also known as ABA 8’-hydroxylase); AOG, ABA β-glucosyltransferase; PYL, abscisic acid receptor PYR/PYL family; PP2C, protein phosphatase 2C; SNRK2, serine/threonine-protein kinase SRK2; ABF, ABA responsive element binding factor. “/” denotes that no significant difference was observed.

GA_20_ is an intermediate in GA biosynthesis with no hormonal activity (Hedden, 1999). The content of no other GAs varied among BT, NT and HT (**Figure 4**). Therefore, GAs were not considered regulators of anthocyanin biosynthesis, and related DEGs were not analyzed.

### 3.8 qRT−PCR validation of differentially expressed genes

**Figure 6:**
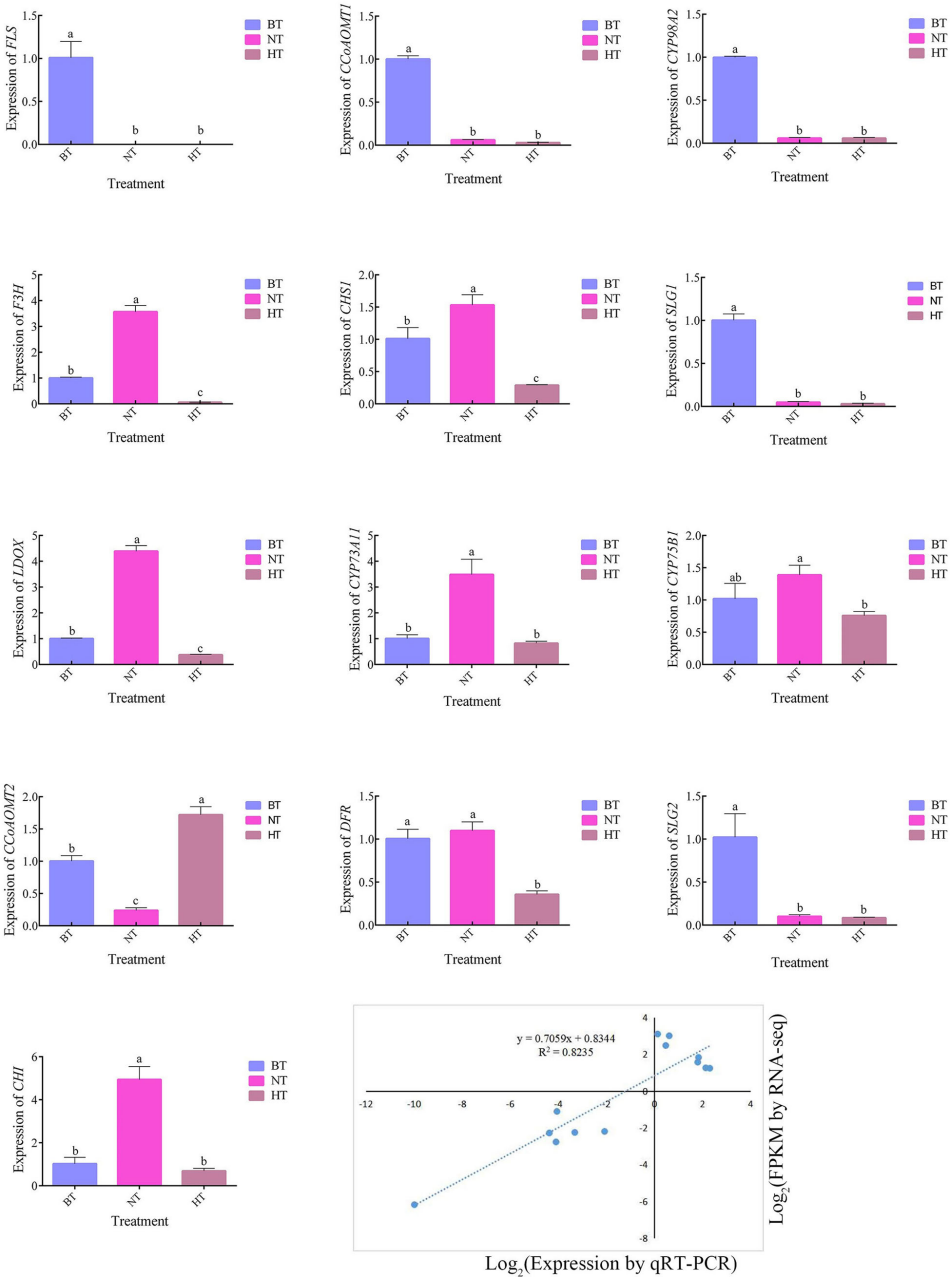
The genes of the anthocyanin and flavonoid synthesis pathways in the transcriptome were verified by qRT−PCR. BT was set as the reference group for each temperature treatment, in which the gene expression level was recorded as 1. The different letters indicate significant differences among different treatments according to Duncan’s test (p < 0.05).

To validate the reliability of the expression profiles obtained from RNA-Seq, 13 DEGs related to flavonoid biosynthesis and anthocyanin biosynthesis were selected for quantitative real-time PCR (qRT−PCR) (**Figure 6**). The results of the qRT−PCR analysis of genes in the anthocyanin biosynthetic pathway were consistent with the RNA-seq results. The expression levels of *CHS, F3H* and *DFR*, key genes in anthocyanin biosynthesis, were upregulated in NT but downregulated in HT. These results indicate that the RNA-seq data were reliable for assessing the transcriptomic changes induced by high temperature.

## 4 Discussion

### 4.1 High temperature inhibits the activation of anthocyanin biosynthesis in sweet cherry peels

Sweet cherry presents high environmental requirements during coloring. An unfavorable environment leads to the inhibition of anthocyanin accumulation, which directly affects the appearance and commercial value of fruits (Esti et al., 2002). Anthocyanin biosynthesis is affected by various external factors, such as temperature and light. Low temperature promotes the expression of anthocyanin biosynthetic pathway genes and increases anthocyanin contents (Steyn, 2009; Ubi et al., 2006), while high temperature affects the activity of related enzymes (Mori et al., 2005) and inhibits the accumulation of anthocyanins and glycosides (Ban et al., 2007). Global warming and inconsistent temperature conditions are leading to serious implications for the coloring of sweet cherry fruits. Therefore, elucidating the molecular mechanism of temperature-regulated anthocyanin accumulation is important.

In this research, high temperature severely inhibited anthocyanin accumulation in sweet cherry peel and delayed the coloring process (**Figure 1**). This phenomenon is similar to that in apple (Lin-Wang et al., 2011), grape (Yamane et al., 2006) and eggplant (Lv et al., 2019). Transcriptome and qRT−PCR analyses showed that the general expression of structural genes involved in anthocyanin biosynthesis was significantly upregulated under normal temperature but changed little under high temperature (**Table 1**). The bHLH transcription factor is an essential component of the MYB-bHLH-WD40 complex that directly regulates the expression of anthocyanin biosynthesis-associated structural genes (Jaakola, 2013). The expression of 3 *bHLH* genes was also found to be downregulated under high temperature compared with normal temperature (**Table 1**). Among them, *bHLH13* was annotated encoding the N-terminal and helix-loop-helix DNA-binding domains of bHLH-MYC and R2R3-MYB transcription factors in the Pfam database. These data indicated that high-temperature high temperature inhibited the activation of the anthocyanin biosynthesis pathway in sweet cherry peel during fruit development and finally led to slow coloring.

### 4.2 Roles of plant hormones in the coloring inhibition induced by high temperature

Plant hormones are essential regulators of anthocyanin biosynthesis in fruits. ABA plays an important role in the ripening and coloring of nonclimacteric fruits, such as strawberry, sweet cherry and grape (Setha et al., 2005; Jia et al., 2011; Wang et al., 2022). ABA treatment significantly induced anthocyanin accumulation in sweet cherry fruits, while treatment with an ABA biosynthesis inhibitor blocked anthocyanin production (Ren and Leng, 2010; Shen et al., 2014). In this research, high temperature severely inhibited the increase in ABA levels in sweet cherry peel (**Figure 4**). More genes involved in ABA signal transduction were upregulated in NT than in HT (**Table 2**). The ABA level showed a significant correlation with the anthocyanin level (**Supplementary Material S3**). This finding indicates that ABA is a key regulator in the high-temperature-inhibited anthocyanin biosynthesis in sweet cherry peel.

Further RNA-seq showed that HT changed the expression of several genes related with ABA metabolsim. *BCH* and *ZEP* encode enzymes, but not limiting enzymes, in ABA biosynthesis (Hieber et al., 2000; Xia et al., 2022). The expression levels of them were only slightly lower in HT than in NT with the log_2_(fold change) of no more than -1.5 (**Table 2**). Therefore, the downregulation of *BCH* and *ZEP* might have little effects on ABA accumulation. *CYP707A* and *AOG* encode key enzymes in the catabolism and inactivation of ABA, respectively (Xu et al., 2002; Saito et al., 2004). The expression of the two genes was much higher in HT than in NT and lower in NT than in BT (**Table 2**). This indicates that the expression of *CYP707A* and *AOG* decreased during fruit coloring, while high temperature strongly hindered the process. These data show that the regulation of ABA catabolism and inactivation rather than ABA biosynthesis was responsible for the high temperature-inhibited ABA accumulation. The study on detailed effects of high temperature on ABA metabolism in sweet cherry peel is still needed in subsequent studies.

Previous studies showed that GA mainly exerted a negative role in the ripening and coloring process of sweet cherry. GA_3_ application delays ABA accumulation, fruit size increase and ripening, reduces anthocyanin levels and produces fruits with less color (Kondo and Danjo, 2001; Usenik et al., 2005; Kuhn et al., 2021). GA_4_ is also significantly and inversely correlated with the ripening parameters of sweet cherry (Teribia et al., 2016). In this research, only the content of GA_20_ was observed to be significantly correlated with the anthocyanin level in sweet cherry peel (**Supplementary Material S3**). However, GA_20_ is an intermediate in GA biosynthesis and has no hormonal activity (Hedden, 1999). Therefore, GA could be inferred to be not related to high-temperature-inhibited anthocyanin biosynthesis.

Other plant hormones, such as IAA, cytokinins and JA, are also effective in regulating anthocyanin biosynthesis (Shan et al., 2009; An et al., 2015; Ji et al., 2015). However, related studies in sweet cherry are still scarce. In this research, although the levels of IAA, cZ, cZR, JA and SA in sweet cherry peel differed between NT and HT (**Figure 4**), they showed no significant correlation with the anthocyanin level (**Supplementary Material S3**), indicating their minor role in high-temperature-inhibited anthocyanin biosynthesis.

### 4.3 Roles of sugar and acid in the coloring inhibition induced by high temperature

Sugar is the precursor of anthocyanin (Smeekens, 2000). The metabolism and accumulation of sugar in fruits is affected by environmental and ecological factors (Kerepesi et al., 2000; Watanabe et al., 2000; Moustakas et al., 2011). In Arabidopsis, anthocyanin production in cotyledons or leaves increases when seedlings are grown in sugar-containing medium (Mita et al., 1997; Ohto et al., 2001). Sucrose is closely related to anthocyanin biosynthesis (Hara et al., 2004; Zhang et al., 2022). It acts as an endogenous trigger that modulates the expression of anthocyanin biosynthesis genes in grape berry skins (Boss et al., 1996). In Arabidopsis, exogenous sucrose increased the transcript levels of several structural genes in anthocyanin biosynthesis by several hundred-fold (Solfanelli et al., 2006) and showed a synergistic effect with ABA on anthocyanin accumulation (Loreti et al., 2008).

In this research, high temperature suppressed the accumulation of glucose, fructose, sorbitol and galactose in sweet cherry peel but not sucrose (**Figure 3**). Fruit sugars mainly come from the photosynthetic product of leaves. It was reported that 35°C/25°C inhibited the photosynthetic rate of ‘Satohnishiki’ sweet cherry compared to 25°C/15°C condition (Beppu et al., 2003). Therefore, the suppressed sugar accumulation could be resulted from the inhibition of photosynthesis by high temperature. Sorbitol is the major photosynthetic product as well as the major sugar transported to fruits in cherry (Roper et al., 1988). It is largely converted to fructose or glucose by sorbitol dehydrogenase (SDH) upon reaching fruits (Aguayo et al., 2013; Zhang et al., 2014). In this research, no DEGs were identified encode any sorbitol transporters, but the expression of two *SDH-like* genes were upregulated in HT than in BT and NT (**Supplementary Material S5**). Two genes respectively encoding glucan endo-1,3-β-glucosidase (GEBG) and β-glucosidase (β-Glu) were also upregulated in HT than in NT. GEBG and β-Glu participate the generation of glucose from glucan and cellulose, respectively. The upregulation of the 4 genes above are all related to the generation of fructose or glucose, and could be a response of sugar deficiency in fruits of HT. Conversely, the expression of a gene encoding invertase (INV) was down regulated in HT than in NT. INV catalyze the hydrolysis of sucrose to glucose and fructose. The inhibition of *INV* expression matches the relative stable sucrose level in HT in condition of lower total sugar (**Figure 3**).

Despite the significant difference of sugar levels no significant correlation was detected between any sugar and anthocyanin (**Supplementary Material S3**), indicating that lower sugar content is not a main cause of the high-temperature-inhibited anthocyanin biosynthesis. However, that sugar metabolism contributed to the process as a synergistic factor of ABA is still a possibility. Sucrose is a minor component of fruit sugar in sweet cherry, accounting for no more than 2% of the total soluble sugar. It may not be important in the regulation of anthocyanin biosynthesis.

Most studies exploring the relationship between acid and anthocyanin are focused on the effect of pH on anthocyanin stability and enzyme activity (Lou et al., 2019). A given pigment can exhibit different colors under different pH conditions (Fossen et al., 1998). The total acid content in sweet cherry peel increased and the pH decreased under both high temperature and normal temperature. Although the expression of a gene encoding NADP-malic enzyme (NADP-ME) was lower in HT than in NT (**Supplementary Material S5**), no significant difference of total acid and pH was detected between the two treatments (**Figure 3**). Therefore, acid metabolism should not be related to the high-temperature-induced coloring inhibition of sweet cherry fruits.

**Figure 7:**
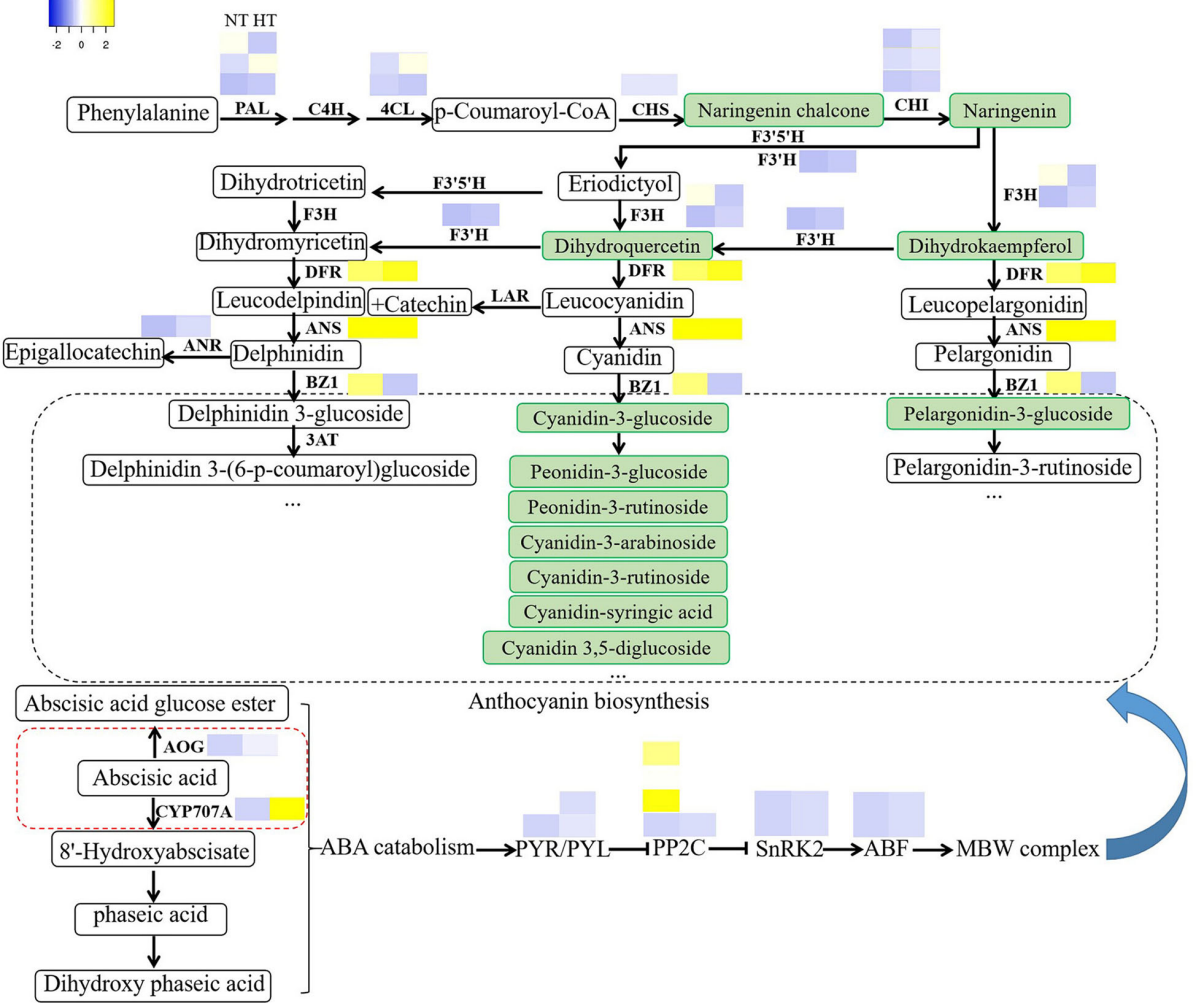
High temperature affects anthocyanin biosynthesis through the ABA catabolic pathway. DEGs from anthocyanin biosynthesis and ABA catabolism pathway genes and selected TFs were visualized as a heat map based on log_2_-fold changes obtained from high temperature treatments against the normal temperature treatment.

Overall, our results indicated that high temperature severely inhibits the coloring of sweet cherry. ABA, an important positive regulator of the ripening and coloring of nonclimacteric fruits, probably plays a key role in the process. High temperature delays the downregulation of *CYP707A* and *AOG* expression during fruit coloring and leads to higher ABA catabolism and ABA inactivation (**Figure 7**). This causes a lower ABA level in fruit peel and delays coloring.

## Data availability statement

The datasets presented in this study can be found in online repositories. The names of the repository/repositories and accession number(s) can be found in the article/**Supplementary Material**.

## Author contributions

HW conceived and designed the experiments. YT and BW conducted the experiments, collected the data and wrote the manuscript. LX and XZ collected the data and performed the statistical tests. GW, YS and HW read and revised the manuscript. All authors contributed to the article and approved the submitted version.
