# Supplementary material for: High temperature inhibited the accumulation of anthocyanin by promoting ABA catabolism in sweet cherry fruits
Source: Front Plant Sci. 2023 Feb 13;14:1079292. doi: 10.3389/fpls.2023.1079292 (PMC9968857; doi:10.3389/fpls.2023.1079292)
Supplement: Supplementary file 1 [file Table_1.docx]

Supplementary Material

# Supplementary Data

**Supplementary Material S1.** The primers used for real-time qPCR

| Gene name | Primer | Squence(5’-3’) |
| --- | --- | --- |
| Actin | F | AATGGTGAAGGCTGGATTTG |
|  | R | ACATCCTTCTGACCCATAC |
| PavFLS | F | CATGTCGTGGGCTGTTTTCG |
|  | R | CCGGTGTCGGTATTCAGCAT |
| PavCCoAOMT1 | F | GGCAGACAGGCGACTATTGA |
|  | R | TCCCATCACCCAAAGAAGCA |
| PavCYP98A2 | F | TGGGCTCTAGCTGAAGGAGT |
|  | R | TGCACAAGTGCGAATGAAGC |
| PavF3H | F | AGAGGGGCCAATCACCTACA |
|  | R | CATCGGCTGACTTGGTCTCC |
| PavCHS1 | F | AACATGTCGAGTGCCTGTGT |
|  | R | AAACAGCACACCCCAATCCA |
| PavSLG1 | F | TTTGGATGGGGAAAGCCATCA |
|  | R | GTCCTTCAAAGTCAAGCGCA |
| PavLDOX | F | CGCCAATCTTCCCACCAAGA |
|  | R | TGTTGAGCAGAGCCTCTTGAC |
| PavCYP73A11 | F | TGAGGCCAATGGCAATGACT |
|  | R | TGATCCCAAGGATTGGCAGG |
| PavF3’H | F | TAATGGCTGCAACCCTGGTC |
|  | R | AGGTGAGCCCATATGCTTCG |
| PavCCoAOMT2 | F | AAGAGGTTGGCCACAAGAGC |
|  | R | TGCCATCGCCAATTCTCTGA |
| PavDFR | F | TGACGGACCAAGGGTTTGAG |
|  | R | GGTGTTGTCGTCAGCCTCAT |
| PavSLG2 | F | CATGAGTGATGGCGTAGGCA |
|  | R | GCAGATCCTCATTGCGTTCA |
| PavCHI | F | CGGAGGCAGTTCTAGAGTCG |
|  | R | TTTCCCATTTCCGGCCTCAT |
