# Supplementary material for: High temperature inhibited the accumulation of anthocyanin by promoting ABA catabolism in sweet cherry fruits
Source: Front Plant Sci. 2023 Feb 13;14:1079292. doi: 10.3389/fpls.2023.1079292 (PMC9968857; doi:10.3389/fpls.2023.1079292)
Supplement: Supplementary file 3 [file Table_3.docx]

Supplementary Material

# Supplementary Data

**Supplementary Material S3.** Correlation coefficients between anthocyanin content and other physiological parameters. * denote that the correlation is significant at the level of 0.05.

|  | Anthocyanin |  | Anthocyanin |
| --- | --- | --- | --- |
| Glucose | 0.961 | ABA | 0.999* |
| Fructose | 0.964 | IAA | 0.985 |
| Sorbitol | 0.807 | cZ | -0.129 |
| Galactose | 0.736 | cZR | -0.180 |
| Sucrose | 0.790 | GA_1_ | 0.212 |
| Total sugar | 0.909 | GA_20_ | 1.000* |
| Total acid | 0.498 | JA | -0.564 |
| pH | -0.900 | SA | 0.742 |
