# Supplementary material for: High temperature inhibited the accumulation of anthocyanin by promoting ABA catabolism in sweet cherry fruits
Source: Front Plant Sci. 2023 Feb 13;14:1079292. doi: 10.3389/fpls.2023.1079292 (PMC9968857; doi:10.3389/fpls.2023.1079292)
Supplement: Supplementary file 4 [file Table_4.docx]

Supplementary Material

# Supplementary Data

**Supplementary Material S4.** Statistics of differentially expressed genes

| Group | DEG Number | Upregulated | Downregulated |
| --- | --- | --- | --- |
| BT vs NT | 1954 | 984 | 970 |
| BT vs HT | 1781 | 931 | 850 |
| NT vs HT | 1005 | 424 | 581 |
