# Supplementary material for: High temperature inhibited the accumulation of anthocyanin by promoting ABA catabolism in sweet cherry fruits
Source: Front Plant Sci. 2023 Feb 13;14:1079292. doi: 10.3389/fpls.2023.1079292 (PMC9968857; doi:10.3389/fpls.2023.1079292)
Supplement: Supplementary file 5 [file Table_5.docx]

Supplementary Material

# Supplementary Data

**Supplementary Material S5.** DEGs involved in sugar and acid metabolism

| Pathway | Gene name | Gene ID | Log_2_(fold change) | | |
| --- | --- | --- | --- | --- | --- |
|  |  |  | BT vs NT | BT vs HT | NT vs HT |
| Sorbitol conversion | *SDH-like* | gene_Pav_sc0002475.1_g320.1.br | / | 2.37 | 2.98 |
|  | *SDH-like isoform X1* | gene_Pav_sc0002475.1_g330.1.br | / | 2.75 | 2.91 |
| Glucoside hydrolysis | *GEBG 5 isoform X1* | gene_Pav_sc0001258.1_g760.1.mk | / | / | 1.15 |
|  | *β-Glu 44-like* | gene_Pav_sc0000103.1_g440.1.mk | -1.62 | / | 1.87 |
| Sucrose hydrolysis | *INV* | gene_Pav_sc0000370.1_g250.1.mk | 2.45 | 1.41 | -1.05 |
| Malic acid conversion | *NADP-ME* | gene_Pav_sc0000212.1_g790.1.mk | / | / | -1.05 |

Note: SDH, *sorbitol dehydrogenase*; GEBG, glucan endo-1,3-β-glucosidase; β-Glu, β-glucosidase; INV, intervase (β-fructofuranosidase); NADP-ME, NADP-dependent malic enzyme.
